# Lab-In-Syringe with Bead Injection Coupled Online to High-Performance Liquid Chromatography as Versatile Tool for Determination of Nonsteroidal Anti-Inflammatory Drugs in Surface Waters

**DOI:** 10.3390/molecules26175358

**Published:** 2021-09-03

**Authors:** Celestine Vubangsi Gemuh, Burkhard Horstkotte, Petr Solich

**Affiliations:** Department of Analytical Chemistry, Faculty of Pharmacy in Hradec Králové, Charles University, Akademika Heyrovského 1203, 500 05 Hradec Králové, Czech Republic; gemuhc@faf.cuni.cz (C.V.G.); Solich@faf.cuni.cz (P.S.)

**Keywords:** Lab-In-Syringe, Lab-On-Valve, bead injection, nonsteroidal anti-inflammatory drugs, water analysis, online coupling, high-performance liquid chromatography

## Abstract

We report on the hyphenation of the modern flow techniques Lab-In-Syringe and Lab-On-Valve for automated sample preparation coupled online with high-performance liquid chromatography. Adopting the bead injection concept on the Lab-On-Valve platform, the on-demand, renewable, solid-phase extraction of five nonsteroidal anti-inflammatory drugs, namely ketoprofen, naproxen, flurbiprofen, diclofenac, and ibuprofen, was carried out as a proof-of-concept. In-syringe mixing of the sample with buffer and standards allowed straightforward pre-load sample modification for the preconcentration of large sample volumes. Packing of ca. 4.4 mg microSPE columns from Oasis HLB^®^ sorbent slurry was performed for each sample analysis using a simple microcolumn adapted to the Lab-On-Valve manifold to achieve low backpressure during loading. Eluted analytes were injected into online coupled HPLC with subsequent separation on a Symmetry C18 column in isocratic mode. The optimized method was highly reproducible, with RSD values of 3.2% to 7.6% on 20 µg L^−1^ level. Linearity was confirmed up to 200 µg L^−1^ and LOD values were between 0.06 and 1.98 µg L^−1^. Recovery factors between 91 and 109% were obtained in the analysis of spiked surface water samples.

## 1. Introduction

Nonsteroidal anti-inflammatory drugs (NSAIDs) are known for their effectiveness in treating pain, fever, and inflammation. Moreover, it has been put forward that these drugs have some anti-cancer activity [1,2]. NSAIDs can be purchased without prescription and are also used in livestock breeding [3] so they are some of the most consumed drugs today. Apart from improper disposal of outdated drugs via domestic waste and drainage, the biotransformation of NSAIDs is only partial [4,5]. In fact, elimination rates in wastewater treatment plants reach only around 60% for diclofenac (DCF) and 70–90% for ibuprofen (IBU) and naproxen (NAP) [6,7], so they can be found in microgram per liter concentrations in effluents and receiving surface waters [6,8].

The accumulation of NSAIDs can have severe adverse effects on aquatic organisms and be toxic to various animals [9]. For these reasons, DCF was included in the watchlist of substances in the EU (2013/29/EU directive) [10] and DCF, IBU, and NAP were classified as emerging organic pollutants.

Today, monitoring data for NSAIDs have improved widely; as a result, DCF was removed from the watchlist in 2018 [5,8]. However, there is still a need for improved analytical methodologies capable of monitoring including required sample preparation [11,12]. Such methods can greatly benefit from analyte preconcentration to achieve the required sensitivity as well as for matrix removal to enhance the reliability of posterior analyte separations by HPLC or CE [13]. Apart from online solid-phase extraction (SPE), i.e., using a prefilled sorbent cartridge on the injection loop on HPLC, most sample preparation procedures are carried out manually, implying additional time, effort, and possible errors. On the other hand, online SPE is limited by the loadable volume and the need for regular sorbent exchange. Preferentially, HPLC with mass spectrometry (MS) has been used for NSAID analysis due to the higher sensitivity and selectivity [5], while this technique also generally requires, mostly sorbent-based, sample clean-up.

Sample pre-treatment represents a major challenge in the analytical process, particularly for the determination of pollutants in environmental samples and complex matrices. Moreover, this step is a bottleneck in terms of sample processing speed and has a significant impact on the result due to possible sample contamination, analyte loss, or random deviations in sample handling that can add up to a significant error. Consequently, constant improvement of sample preparation approaches in terms of speed, reliability, clean-up, and analyte enrichment is of paramount interest.

SPE has been widely used in NSAID analysis in various matrices. Recent developments include novel sorbents and methodologies such as molecularly imprinted polymers [14,15], dispersive magnetic SPE [16,17], stir-bar sorptive extraction [18], rotating disk sorptive extraction [19], online SPE based on a deep eutectic solvent polymer [20], sol–gel hybrid material [21], magnetic metal–organic framework nanocomposites, polyaniline-coated nanofibers [22,23], or nickel–iron layered double hydroxide [24]. Despite their proven efficiency, these procedures are manual, and often laborious and time-consuming sorbent synthesis and characterization by NMR, electron microscopy, or FT-IR are required.

On the other hand, cartridge-based SPE using commercial hydrophilic–lipophilic balanced (HLB) sorbents has proven to be among the most reliable procedures and the most often used materials to preconcentrate pharmaceutical contaminants from waters [5,25,26,27,28]. Here, automation of the sample pre-treatment step still plays a minor role despite the attractive benefits offered, such as a lower risk of sample alteration during processing and a general gain in reproducibility. Moreover, automation fits the vision of green chemistry by achieving miniaturized methodologies that require less solvent and sorbent, i.e., reducing waste, and that can increase the overall sample throughput [29,30].

The bead injection (BI) approach is a powerful approach for the automation of SPE procedures [31]. By handling the sorbent as a suspension, a microcolumn of sorbent particles is packed in a flow channel by trapping the “beads” contained in a certain volume of the suspension. Then, the sample is perfused through the bead packing, i.e., sorbent loading, for analyte retention. Likewise, sorbent conditioning, washing to eliminate matrix remains, and analyte elution are performed. After usage, microcolumn removal is done by sorbent re-aspiration and discharge. Only a fraction of the sorbent amount of what holds commercial cartridges is usually required and manual intervention is unessential [30,31].

BI is typically performed inside a purpose-adapted flow conduit serving as the stator of a selection valve leading to a flow technique, denoted, similarly to the flow conduit itself, Lab-On-Valve (LOV) [32], derived from sequential injection analysis [33]. Straight and smooth flow channels and transparency facilitate bead handling and the observation of bead trapping during method optimization. For BI, sorbents of soft nature (sephadex, sepharose, cellulose) and of diameters > 50 µm of a variety of possible functionalities are generally preferred as they are ideal for reproducible and trouble-free handling [34].

Here, we decided on a hydrophilic–lipophilic balanced sorbent of 30 µm particle diameter, following a previous, yet far more complex BI approach [28]. Thus far, BI-LOV has proven versatile for SPE of insecticides [35], food additives [36], metals in various matrices [37,38,39], and polychlorinated bisphenyls [40], among others.

As in the parental technique, sequential injection analysis, solution mixing in the LOV manifold is achieved via zone stacking and dispersion in the tubing manifold. However, for sample volumes larger than a few hundred microliters, i.e., aiming for higher loading volumes and preconcentration, zone mixing is inefficient for in-system modification of the sample, e.g., with a loading buffer. Consequently, LOV configurations have been proposed that integrate a confluence in the holding coil and that use two individual pumps to enable in-line, pre-load sample modifications [28,39,41].

In this work, we explore the combination of LOV-BI with the flow-batch technique Lab-In-Syringe (LIS) [42,43] as a proof-of-concept. LIS, similar to LOV originating from sequential injection analysis, is an ideal tool to automate and miniaturize liquid-phase microextraction approaches or downscale chromogenic assays [43,44]. The typical flow manifold is replaced by the syringe void itself, into which all solutions can be aspirated step-by-step as required and mixed homogenously by magnetic stirring [45]. In this way, the operation and characteristics of LOV and LIS are opposed and complement each other [46].

Recently, in-syringe dispersive SPE was described [47], while the potential of combining LIS with BI-SPE has not been explored yet. Using the syringe pump as a mixing chamber for sample and loading buffer, i.e., taking advantage of large-volume mixing as typical for LIS, with subsequent LOV-housed BI would thus allow an increased volume of sample to be treated. The resulting system was coupled online to HPLC to study the LIS–LOV automated BI-µSPE as an automated front-end sample pre-treatment for HPLC. Five NSAIDs were taken as model analytes, namely ketoprofen (KET), flurbiprofen (FLB), NAP, DCF, and IBU, and determined as contaminants in surface waters. To the best of our knowledge, the hyphenation of the LIS and LOV concepts has not been reported yet.

## 2. Results

### 2.1. System Setup and Preliminary Considerations

Typically, sorbents of particle sizes of >100 µm are used for BI due to the resulting low flow resistance. On the other hand, many SPE sorbents have particle diameters around 30 µm, thus involving also a larger active surface. A modification in our system to deal with this problem was the usage of an adaptor to enable the creation of an SPE column of slightly wider diameter than the LOV flow channels and, in particular, with a highly permeable frit to trap and use sorbent beads of small diameter without facing backpressure problems.

The additional solvent filter served to avoid the blockage of possibly escaping sorbent beads that would be injected into the high-pressure line. However, by changing the membrane filter regularly, no such issue was observed, indicating that the featured low-pressure frit was reliable and suitable for our purposes.

Often, the sample needs to be adjusted before SPE, such as by dilution, to decrease its salinity or solvent content or to adapt its pH value. The task of mixing a large volume of sample (>1 mL) with a much smaller volume, e.g., of a buffer, in a flow system requires some considerations as zone penetration is limited by the small tubing cross-section areas. Typically, the task is done by implementing a mixing chamber into the flow manifold, by multiple alternation of solution aspiration and mixing the stacked zoned by dispersion, or by the confluent addition of the secondary solution. For LOV, various modes to implement a second pump for confluence mixing have been proposed [36,37,38]. Here, we combined for the first time LOV with bead injection with the LIS operation principle, i.e., the sample and loading buffer were homogenously mixed inside the syringe void by magnetic stirring before loading, which enabled preconcentration from volumes as large as 4 mL at once.

### 2.2. Experiments on the Combined System Using UV Detector

#### 2.2.1. Study of Sorbent, Loading Conditions, and Eluent Composition

Two operation modes were considered for the preconcentration of NSAIDs. The first one was preconcentration of the analytes in their anionic form in moderately alkaline conditions with posterior elution using an acidified mixture of an HPLC-compatible solvent with water. The second was preconcentration of the undissociated analytes in a moderately acidic medium, thus using hydrophobic interaction as the main SPE principle, with posterior elution also using a water–solvent mixture with supposedly higher solvent content as in the previous approach and potentially alkalinized.

Screening experiments were performed to choose the better performing mode using NAP, DCF, and KET as analytes. The experiments were performed without injection to HPLC to circumvent the difficulty of finding the optimal volume to load the injection valve for each resin and elution condition, i.e., heart-cut of the eluent zone showing the maximum analyte concentration. Instead, UV spectrophotometric detection in-LOV was carried out using, for this purpose, the integrated multifunctional detection cell, as described in Section 2.2 and Section 4.2. 

For each mode, a polymer resin of high loading capacity was tested, with Strata X-AX as the sorbent with anionic exchange and hydrophobic interaction and Oasis HLB as the sorbent with hydrophobic interaction as well as the possibility of hydrogen bonding with the analyte. The sorbent beads were trapped in the flow channel on LOV position 2 leading to the integrated detection cell. A flow rate of 10 µL s^−1^ was chosen, which was low enough to avoid excessive overpressure by the microcolumn.

While the general behavior and signal heights were comparable for both resins, significantly higher reproducibility was found using the Oasis HLB resin (Figure S1 in Supplementary Materials), i.e., a geometric mean of RSD values of 7% and 10% at 80% (*v*/*v*) and 60% (*v*/*v*) methanol (MeOH) in the analytes versus 29% and 19% for the Strata X-AX resin using acidified methanolic eluents of the same concentrations. In consequence, the HLB resin was chosen for further investigation. As expected, eluent alkalinization did increase the peak heights for a lower concentration of MeOH but also decreased the reproducibility of the signal height studying elution without HPLC and was therefore omitted.

The addition of 10 mmol L^−1^ ammonium hydroxide to the eluent led to an increase in peak signals for contents lower than 60% (*v*/*v*) MeOH due to analyte dissociation. However, in this way, efficient analyte retention onto the head of the separation column, an imperative condition for the injection of a large volume of eluate for a gain of sensitivity, would have been difficult to achieve. Moreover, the usage of ammonium buffer could result in separation column damage even if a strongly buffered, acidic mobile phase had been used. Finally, higher reproducibility was also achieved by omitting eluent alkalinization (see Figure S2).

Based on these findings, the HLB resin was chosen as the better option in terms of reproducibility and method reliability but omitting ammonium addition to the eluent. On the other hand, this meant abandoning the objective of orthogonality of SPE and HPLC separation.

In terms of sorbent quantity, we found an equal response for sorbent suspension volumes of 200 µL to 400 µL while the system backpressure increased significantly, which diminished the loading reliability and reproducibility. For this reason, a sorbent suspension volume of 200 µL was chosen.

#### 2.2.2. Separation Method for Online Coupling

The effect of mobile phase composition and its effect on the peak shapes and resolution was evaluated on the chosen RP Symmetry C18 column using an injection volume of 20 µL. A pH of 3.5 to avoid analyte dissociation was established with ammonium formate buffer in all experiments. Using a buffer concentration of 25 mmol L^−1^, pH 3.5, and varying the percentage of acetonitrile (ACN) in the range of 20% to 50% yielded poor separation performance and peak shapes. Therefore, partial replacement of ACN by MeOH was tested with a total organic content of 40 to 60%(*v*/*v*), finding that a mobile phase composition of ACN:MeOH: ammonium formate buffer (25 mmol L^−1^) of 30:30:40%(*v*/*v*) at pH 3.5 ensured high peak symmetry and baseline separation of all the analytes within 15 min at 1 mL min^−1^. To decrease the separation time, the flow rate was increased to 1.2 mL min^−1^ with only a slight increase in plate heights (12.7% on average), which allowed peak separation within 13 min.

Finally, the effect of buffer concentration and pH was evaluated in the ranges of 50 mmol L^−1^ to 200 mmol L^−1^ and pH 2.5 to 3.8, respectively. These changes did not show any significant effect on the separation performance or on reproducibility, and the RSD values of the retention times of the analytes were generally <1%. Under these conditions, the analytes eluted in the order KET, NAP, FLB, DCF, and, lastly, IBU.

Connecting the LIS–BI–LOV system to HPLC, it was found that, when using 80% (*v*/*v*) MeOH as eluent, both peak shape and peak resolution on HPLC were unacceptable. Moreover, due to the relatively high viscosity of MeOH–water mixtures, the use of a lower content of acetonitrile rather than a high content of MeOH was favored. A study was performed to determine whether, with an eluent of 50% (*v*/*v*) acetonitrile, being the highest concentration that allowed sample loading on HPLC without significant peak deterioration, a similar elution efficiency could be achieved as with 80% (*v*/*v*) of MeOH. In effect, 50% (*v*/*v*) acetonitrile yielded equal reproducibility, comparable signal heights, but also improved separation performance. It was therefore chosen for all following experiments.

#### 2.2.3. Sample Volume

In the following, experiments were carried out with the sample preparation system coupled online to HPLC via the injection valve acting as an interface.

The dependency of the analyte signals on the used sample volume was studied in the range of 1 mL to 4 mL. The respective volume of sample was aspirated into the syringe and acidified with 200 µL of 100 mmol L^−1^ hydrochloric acid. The actual volume of acidified sample loadable onto the in-system packed microcolumn was smaller by 400 µL, i.e., the volume of the holding coil, thus varying between 0.8 and 3.8 mL (corresponding to 0.67 to 3.62 mL of the genuine sample).

**Figure 1:**
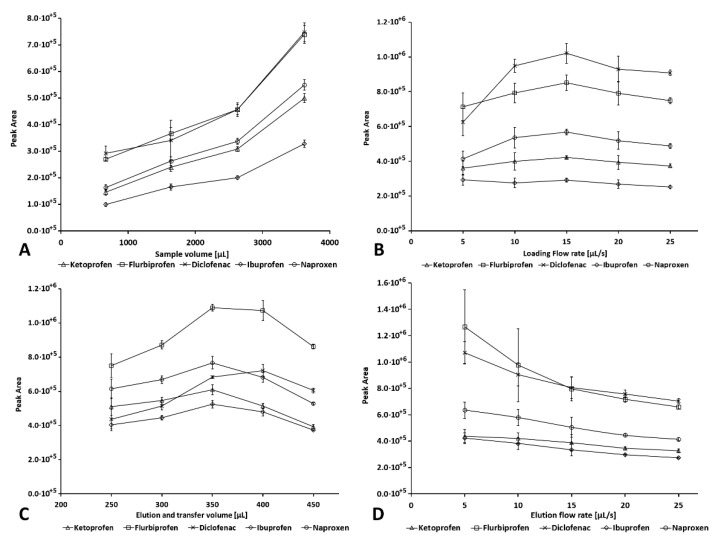
Studies of parameter effects for method optimization. Signals of NAP were scaled by factor 0.1. (**A**) Sample volume. (**B**) Loading flow. (**C**) Elution flow rate. (**D**) Effect of the transfer volume on the peak area at online coupling of LIS–LOV–SPE and HPLC. Conditions other than stated in Section 2: (**A**) Loading of mixed 0.2 ppm standard acidified with 200 µL of 100 mmol L^−1^ hydrochloric acid at 15 µL s^−1^. Microcolumn of ca. 5 mg HLB sorbent, injection of 220 µL of eluent (50% (*v*/*v*) ACN) to HPLC. (**B**) as (**A**) but with 3.8 mL of loaded standard. (**C**) as (**B**) but 15 µL s^−1^ loading speed. (**D**) as (**C**) but with an elution volume of 350 µL.

Peak areas in dependency on the effectively loaded volume and specific conditions are given in Figure 1A. Peak areas were increasing linearly for the sample volume without any observable overload of the microcolumn. While it is probable that an even larger sample volume could be loaded (depending on the concentrations of the analytes), higher volumes were not tested to avoid the need for prolonging the flow procedure beyond the time required for the HPLC separation that was running in parallel to the analyte preconcentration. It is pointed out that even with 4 mL loaded, the resolution of the most critical peaks (ketoprofen and naproxen) was still 1.6 under the chosen separation conditions.

#### 2.2.4. Loading Flow Rate

The effect of the flow rate for sample loading was studied in the range of 5 to 25 µL s^−1^, corresponding to 0.3–1.5 mL min^−1^. Results and specific conditions are given in Figure 1B. A maximum was found at a flow rate of 15 µL s^−1^. The lower signals at reduced flow rates were explained by improper compaction of the sorbent under this condition, while the decrease with a higher flow rate is most likely due to incomplete retention on the SPE packing. The best reproducibility and highest signals were found when using 15 µL s^−1^, which was therefore the flow rate chosen for further work. Problems related to overpressure during loading were not observed at any time.

#### 2.2.5. Elution and Transfer Volume

The volume of eluent was initially fixed to 400 µL, corresponding to the volume of the holding coil. The optimal volume of eluent that had to be passed through the sorbent microcolumn for elution and to transfer the analyte towards the HPCL and finally to load the injection loop was studied in terms of reproducibility and signal height in the range of 250 µL to 450 µL. Results and specific conditions are given in Figure 1C. It was found that the optimal volume for eluting and transferring the analytes from the µSPE column into the HPLC injection loop, i.e., the optimal volume before a heart-cut of the elution profile, was 350 µL, which was consequently used in all subsequent analyses. Since the injection volume was very large (220 µL), the most of the eluted analytes was effectively injected. The peak areas increased to this volume, reaching a maximum between 350 and 400 µL, and decreased for larger volumes. The RSD values were generally between 1% and 5%, while for the smallest volume of 250 µL, values of 9% to 13% were obtained. This experiment also showed that the eluent volume of 400 µL was sufficient for the proposed system and method.

#### 2.2.6. Elution Flow Rate

The effect of the elution flow rate was studied in the range of 5 to 25 µL s^−1^ (0.3–1.5 mL min^−1^). Higher flow rates were not tested under the consideration of possible backpressure problems. Experimental conditions and results are given in Figure 1D. The peak area decreased as the elution flow rate increased, as is typically observed due to an insufficient time for phase equilibrium and diffusion of the eluent into the pores of the sorbent, causing a broadening of the analytes’ elution profiles. On the other hand, significantly better reproducibility was found for the higher flow rates, which, as for the loading flow rates, was likely due to improper compaction of the sorbent bed and possible flow channeling. For these reasons, a flow rate of 15 µL s^−1^ was finally chosen as a compromise between reproducibility and signal height.

#### 2.2.7. Influence of Washing Solution

The type of washing solution to remove interfering matrix remains was studied. To avoid untimely elution of the analyte during this step, a solvent of low elution power is typically used. Here, water with different quantities of MeOH (0 to 20% (*v*/*v*)) was tested. The results are given in Figure S3A, and only marginal signal changes were observed over the studied range. Considering the little effect observed and the better reproducibility using water than for all MeOH–water mixtures, as well as for procedural simplicity, water was chosen as the washing solution.

#### 2.2.8. Loading Acidity

The effect of the concentration of the hydrochloric acid used for sample acidification before loading was studied for 50, 100, 150, and 200 mmol L^−1^. The volume was kept constant to 200 µL so not to deteriorate sensitivity by a larger acid volume. Results and experimental conditions are given in Figure S3B. Only a slight increase in peak areas of <7% was observed for FLB and DCF for the highest acid concentration, while the effect for the other three analytes was insignificant, so that the original concentration of 100 mmol L^−1^ was kept.

## 3. Discussion

### 3.1. Method Performance

The final experimental parameters of the developed system and optimized method are summarized in Table S1 (Supplementary Materials). Table 1 summarizes the method performance characteristics, which were performed according to the FDA validation guidelines [48]. Calibration curves for the analytes were constructed by preparing calibration solutions at distinct concentration levels: 5, 10, 20, 50, 100, and 200 µg L^−1^. Acceptable linearity was obtained over this concentration range, with R^2^ values greater than 0.994. LOD and LOQ were calculated by spiking samples with minimum concentrations, giving signal-to-noise ratios equal to 3 and 10, respectively. Thus, LOD and LOQ were found to range from 0.06 to 1.98 µg L^−1^ and 0.2 to 6.0 µg L^−1^, respectively, for all analytes. 

The method precision was evaluated by determining the repeatability (intra-day precision) and intermediate precision (inter-day precision). To evaluate the repeatability, a sample spiked at 20 µg L^−1^ was analyzed in sextuplicate (n = 6), and the RSD of the peak areas was calculated (RSD < 8%). For inter-day precision, the same concentration of spiked (20 µg L^−1^) sample was prepared on 3 separate days, analyzed in quadruplicate (n = 12), and the RSD calculated from the peak areas as well (RSD < 10%). Effective signal enhancement was found to range from 14 to 32. It was calculated from peak areas obtained by injecting the eluate from the automated BI–SPE and injections of the same volume (220 µL) of aqueous standard omitting the automated procedure. Considering the injection volume of 20 µL that was used for separation method optimization, the achieved preconcentration factor can be considered 11-times higher. Linearity was confirmed for concentrations between 5 and 200 µg L^−1^ for all analytes. The relatively wide range of the preconcentration factors was due to the use of isocratic elution and differences in the elution profile shape of the individual analytes (see Figure 1C).

In Table S2, our method is compared with formerly reported approaches using, as here presented, spectrophotometric detection, liquid chromatography, and SPE methodologies for sample preparation, in part based on novel sorbent and methodologies.

The achieved analytical performance was comparable to earlier methods and was superior in the following aspects: (i) a commercial sorbent was used, thus not requiring lasting synthesis and assembly of extraction devices or characterization; (ii) the entire method was automated, including, via the bead injection approach, the automated exchange of sorbent; (iii) only a fraction of what is used in standard cartridges was required for each analysis; (iv) the methodology was faster than formerly reported automated approaches as well as most manual procedures, and by parallel operation of separation and sample preparation of the subsequent sample, a sample throughput of >4 h^−1^ was feasible, which could be further increased by gradient HPLC; (v) the sample volume used is similar as in protocols using commercial sorbent cartridges yet rather reduced in comparison to former reports, while repeated loading with sample could be used to enhance the method’s sensitivity as required; and (vi) vice versa, loading of less sample dilution is straightforward to extend the working range of the method without risking overload of the sorbent.

In comparison to other methods, the nominal preconcentration factors were only moderate considering the injected volume. However, the here applied samples would have not permitted direct injection without gradual deterioration of the separation column by the sample matrix, particularly humin substances. Taking, therefore, as a fair comparison the 11-times lower injection volume used during separation optimization, the signal enhancement would be correspondingly larger.

It should be pointed out as well that most methods used a higher sample volume, while, using the proposed method, only 4 mL was required, with 1 mL more for cleaning the connecting tube to the autosampler. However, the loading step could be repeated several times in order to preconcentrate from a larger sample volume, and it would be possible to extend the linear range by in-syringe sample dilution or loading less volume, given the method’s flexibility. For applications to biological samples such as serum samples, the system could be easily downscaled using, for instance, a smaller syringe.

The eluent volume into which the retained analytes are back-extracted can be reduced by drying the sorbents with air to reduce the required injection volume. This would consequently yield a higher preconcentration factor by comparing the achieved peaks to a smaller injection volume of the not-concentrated standard. For reasons of simplicity and considering affected repeatability, this possible operation was not followed.

Compared to an earlier automated method using the same sorbent [28], in-system acidification of the sample was performed, thus reducing the required manual tasks; no post-elution sample dilution was required, and a far simpler instrumental setup and method were required. In particular, although not required in our case, the LIS technique enables in-syringe dilution not only of the sample with buffer solutions for required pH adjustment prior to loading but also with an internal standard to increase the determination reliability by tracing possible uncomplete loading (due to overpressure) or analyte loss by matrix interferents. In the former method, a far higher flow rate was applied for loading, which, in our case, was not studied as the sensitivity decreased for higher flow rates.

In comparison with manually performed SPE protocols, the automation by LIS–BI–LOV as proposed here enables a “just-in-time” sample preparation, including overnight operation, as was done for sample measurements. In contrast to the use of vacuum manifolds, the loading and elution flow rates were highly reproducible and did not depend, as is the case in manual work, on the sample viscosity.

The method is, in principle, applicable also to HPLC with mass spectrometric detection with little adaptations in separation conditions, yet such a detector was unavailable to us. In this case, a significant improvement of the LOD and LOQ values can be expected, yet the aim and outcome of the present work demonstrate—as a proof-of-concept—the hyphenation of the LOV–BI concept and LIS technique and its potential. This is the ability to automated microSPE with sorbent exchange with facilitated large sample pre-load modification.

### 3.2. Sample Measurement

To study the method’s applicability, surface water samples were taken for local rivers and lakes, filtered through hydrophilic polytetrafluoroethylene membrane filters of 0.45 µm pore size. They were analyzed in duplicate, both unmodified as well as spiked with a mixed aqueous standard to a concentration of 20 µg L^−1^. This concentration level, although atypical for assumingly uncontaminated surface waters, was used to avoid artefacts by possible peak overlapping with matrix components, which could not be fully resolved due to the lack of available MS detection. The results of sample analyses are given in Table 2. Average recoveries for KET, NAP, FLB, DCF, and IBU of 105–109%, 100–103%, 99–101%, 91–97%, and 105–107% with relative standard deviations in the range of 4% for surface waters were obtained, respectively. The high analyte recovery thus demonstrates the method’s applicability to water analysis, while the limit of detection would have to be further enhanced by coupling the system for more sensitive detection systems such as mass spectrometry to determine NSAIDs in less contaminated waters. On the other hand, the achieved sensitivity would be sufficient for monitoring higher concentration levels of NSAIDs, as reported for wastewater effluents [8].

## 4. Materials and Methods

### 4.1. Reagents and Solutions

Ultrapure water (18.2 MΩ cm^−1^) provided by a Merck Millipore purifying system (Burlington, Massachusetts) was used throughout. All chemicals used were purchased from Sigma-Aldrich (Merck KGaA, Darmstadt, Germany). MeOH and ACN, used for mobile phase preparation and online SPE, were of HPLC grade. Stock solutions of analytical-grade ammonium hydroxide (2 mol L^−1^), hydrochloric acid, and formic acid were used in the preparation of buffer solutions. Stock solutions of formic acid (20% (*w*/*v*)) and ammonium hydroxide (2 mol L^−1^) were utilized for the preparation of ammonium formate buffer solutions pH 3.5 as an additive to the mobile phase. A solution of 100 mmol L^−1^ hydrochloric acid was used for sample acidification before loading during online SPE. NSAID standards DCF, FLB, IBU, KET, and NAP were obtained from Sigma-Aldrich. Individual stock solutions (10 mg mL^−1^) were prepared in MeOH and preserved at 4 °C in the dark until used. Working standard solutions were prepared daily by appropriate dilution with water. Different sorbent materials were tested for online SPE, which were taken from commercial SPE cartridges including Oasis HLB, 30 µm particle size (Waters GmbH, Etten-Leur, The Netherlands).

Sorbent suspensions of 2.2% (*w*/*w*) were prepared in 50% (*v*/*v*) MeOH. After optimization of the eluent composition, the bead suspension was prepared in 10% (*v*/*v*) can, which ensured improved retention of the analytes and equally high wettability of the sorbent particles.

### 4.2. Flow System

**Figure 2:**
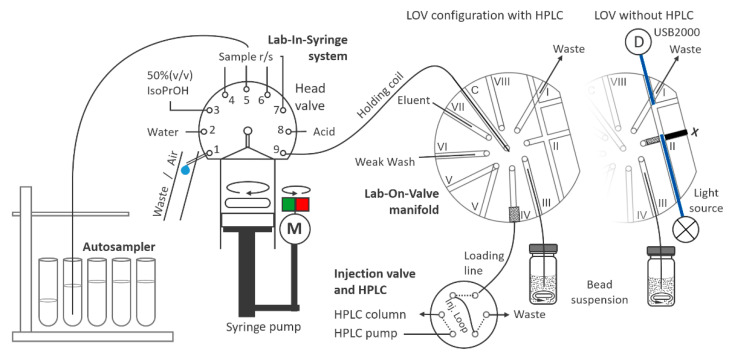
Scheme of Lab-In-Syringe/Lab-On-Valve flow system with the two LOV configurations used. Abbreviations: D—detector, M—motor. Tube dimensions: holding coil 84 cm, 0.8 mm i.d., injection loop 116 cm, 0.5 mm i.d., loading line 32.5 cm, 0.8 mm i.d., line to the bead suspension 13 cm, 0.8 mm i.d.

The combined LIS–LOV system is depicted in Figure 2 with tubing connections and dimensions indicated. Tubings were from fluorinated ethylene propylene of 0.8 mm i.d. if not indicated otherwise. The system consisted of an automatic MicroCSP-3000 syringe pump (FIALab Instruments Inc., Seattle, WA, USA) used for handling all solutions, equipped with a glass syringe of 5000 µL capacity and a low-pressure 8-port selection valve from Vici Valco Inc. (Schenkon, Switzerland).

The syringe pump featured a 9-port ceramic head valve (HV) configured such that HV position 2 was connected to a reservoir of water, position 3 to a cleaning solution of 50% (*v*/*v*) isopropanol, and positions 4 to 7 to individual sample and standard solutions. For sample measurement and calibrations, an AIM 3200 autosampler (AIM Lab, Virginia, QLD, Australia) was connected to HV position 5 to accommodate all standard and sample solutions. HV position 8 was used for an acidifying solution for the SPE loading step, while HV position 1 allowed both aspiration of air as well as syringe content discharge to waste via a draining line.

HV position 9 served for the connection of the syringe pump via a short holding coil to the central port (C) of the secondary low-pressure selection valve. On it, the stator was replaced by a Lab-On-Valve manifold made of Ultem polymer (FIAlab Instruments Inc., Seattle, WA, USA). For clarity, LOV positions are numbered in the manuscript with roman numerals (I–VIII). The LOV integrated a flow-through port on position V and a multipurpose detection flow cell on position II. Position III was connected via a 13 cm long tube of 0.8 mm i.d. to the sorbent beads, which were kept suspended by magnetic stirring at a low rotation speed. Water, washing solution, and eluent were placed on positions VI and VII, respectively, while position I was used for solution discharge to waste. Positions VIII and V were unused and left open. Position V allowed pressure release from the system after microcolumn loading, washing, and analyte elution.

For initial experiments for testing the type of sorbent/loading conditions and eluent composition, the sorbent beads were packed to a microcolumn in the channel on position 2 leading to the detection cell 2. Optical fibers of 1.6 mm o.d., 0.8 mm i.d. (Ocean Optics Inc., Dunedin, FL, USA) enabled spectrophotometric detection with an optical path length of approximately 1 cm. The optical fibers were connected to a miniature USB2000 spectrometer and DH-2000 UV-light source (both Ocean Optics), respectively. One of the fibers was positioned in such a way that channel II was blocked nearly completely, which allowed trapping of the sorbent beads in this channel, as can be seen in Figure 2.

Due to the use of HLB particles of ca. 33 µm, only low flow rates were applicable during initial testing of loading and elution steps in this configuration. When coupling SPE to HPLC, we therefore aimed for packing a microcolumn of wider diameter so as to avoid unacceptable overpressure at higher flow rates and to enable preconcentration of four milliliter of sample in an acceptable time in parallel to HPLC separation. A wider column diameter was featured by a modified commercial 1/8” fitting (Figure S4). A 1/8”-28/” thread was cut, and the inner diameters enlarged. By inserting a circular cut piece of melamine foam, a frit of low flow resistance was obtained. It was sealed and held in place with a silicone ring, cut from a silicone rubber tube (6 mm od, 1.5 mm id, 1 mm height). In this way, a flow chamber that allowed the production of a microSPE column of 2.5 mm in diameter was obtained. The assembly was placed on position 4, facing downwards. Notably, the hydrodynamic pressure decreased with the diameter to the power of four so that the improvement in robustness was significant.

To enable in-syringe mixing of sample and solution for sample acidification, a magnetic stirring bar (14.5 mm length, 3 mm i.d.) was placed inside the syringe void. Rotation was induced by placing a motor close to the syringe with a pile of 6 neodymium magnets fixated on top, which was featured from a pulse width modulated computer fan. The rotation speed was set by a simple analog circuit (scheme given in Figure S5) and controlled by one of three auxiliary terminals of the syringe pump via relay contact. A second one was used to trigger the HPLC pump and data acquisition.

Flow system control and data acquisition during evaluations of sorbent materials and eluent compositions without LC separation were done using FIAlab software wavelengths, choosing 230 nm (for IBU and NAP), 250 nm (for FLB and KET), and 270 nm (for DCF) as analytical wavelengths and 350 nm as a reference wavelength to compensate for the Schlieren effect.

To keep the sorbent particles suspended, a simple holder for magnetic stirring was prepared from a 3D-printed support and a DC motor, equally featured from a computer fan and powered by USB supply (Figure S6). Using a small stirring bar inside the bead suspension and emptying the connecting tube with a plug of air before each aspiration of beads (see Section 4.4), the suspension was of constant composition and homogeneity.

### 4.3. HPLC Instrumentation

The HPLC assays were performed on an isocratic HPLC system (SHIMADZU Handels GmbH, Prague, Czech Republic) consisting of a high-pressure pump (LC-20AD) and a UV/VIS detector (SPD-20A) operating at a wavelength of 230 nm. An RP Symmetry C18 column (WAT045905, 4.6 mm id × 150 mm, particle diameters of 5 µm) from Waters and a C18 OPTI-GUARD^®^ 1 mm guard column (Supelco™ Analytical—Sigma-Aldrich, Merck KGaA, Darmstadt, Germany) were used for the separation of analytes in isocratic mode with a mobile phase of 30:30:40 MeOH:ACN:25 mM ammonium formate, pH 3.5. An 8-port injection valve (Vici Valco, Schenkon, Switzerland) equipped with a high-pressure C2H-2348D Cheminert valve head served as an interface between the combined flow system and the LC instrument equipped with a 220 µL injection loop (Polyether ether ketone, 116 cm, 0.5 mm id) connected to the outlet of port IV. As an additional safety measure, a stainless-steel solvent filter used with 0.45 µm membrane filters (13 mm diameter) was placed between the injection valve and the guard and separation columns.

For HPLC control and data acquisition, the software LabSolutions 5.86 from Shimadzu was used. The flow system was controlled with the software Cocosoft 4.5, kindly provided by Dr. D.J. Cocoví (FITrace group, University of the Balearic Islands, www.fitrace.es [49]).

### 4.4. Method Operation

The optimized operational method for bead injection SPE of NSAIDs coupled online to HPLC is given in Tables S3–S5 in the Supplementary Materials. As the first step, a microcolumn was formed. For this, 200 µL of air was aspirated from LOV position V and expulsed via position III into the bead suspension. This was done to ensure that the respective tube was emptied back into the reservoir and thus to ensure high homogeneity of the suspension and reproducibility of the amount of sorbent aspirated in the next step. The air that remained in the connecting tube also served as a segmentation bubble and so prevented the suspension from entering the syringe void in the next step. The aspirated suspension of 200 µL corresponding to ca. 4.4 mg of beads was then dispensed through channel IV, which was connected to the HPLC injection valve. The beads were retained in the described flow adapter (Figure S1) to produce a short microcolumn of low backpressure.

To preconcentrate the analytes, 4000 µL sample was aspirated into the syringe followed by the aspiration of 200 µL of the buffer and activation of stirring for a few seconds to ensure homogeneous mixing. Thereafter, this mixture was conveyed through the HC and 3800 µL loaded into the microcolumn in the LOV unit at a flow rate of 15 µL s^−1^. Afterward, the syringe was cleaned subsequently with 50% isopropanol and water followed by washing the microcolumn with 300 µL of washing solution aspirated from LOV position VI to remove any sample remains from the microcolumn.

For analyte elution, 400 µL eluent was aspirated from LOV position VII and pushed through the microcolumn at a flow rate of 15 µL s^−1^ into the injection loop to achieve a heart-cut injection. To avoid a significant loss of analyte in this step, particularly during the optimization of the eluent composition and transfer volume, an unusually large injection loop was chosen that was not changed hereafter. Directly after injection and triggering HPLC data acquisition, i.e., in parallel to the running separation, the microcolumn was discarded in a four-step procedure. First, the microcolumn was flushed with 50% (*v*/*v*) isopropanol (the higher viscosity of isopropanol is helpful for the re-suspension of the settled solvent). Second, 50 µL air was aspirated from LOV position V for fluidic segmentation to hinder the beads from entering the syringe void in the next step. Third, the sorbent was aspirated in the prior dispensed 50% (*v*/*v*) isopropanol into the holding coil. Fourth, the sorbent and segmentation bubble were discharged to waste.

For testing the type of resin as well as loading and elution conditions, the LOV-integrated detection cell was used instead of the HPLC system for in-system spectrophotometric detection using the same detection wavelengths. A small piece of melamine foam that was held in place by the optical fibers, as shown in Figure 2, allowed efficient trapping of the sorbent beads in the corresponding channel on selection valve position II. The loaded volumes of the in-syringe buffered sample and eluent were 1200 µL and 400 µL at a flow rate of 10 µL s^−1^.

## 5. Conclusions

In this work, the advantages of the Lab-In-Syringe technique and bead injection methodology were combined. In this way, straightforward handling of milliliter volumes of the sample was enabled and loaded into the on-demand and in-system prepared microcolumns produced from sorbent suspensions. The developed system was coupled online to high-performance liquid chromatography for analyte separation of the eluate. The applicability of the developed system and methodology to the analysis of pharmaceutical contaminants in surface waters was demonstrated, quantitative recoveries of all five analytes were obtained, and signal enhancement ranging from 14 to 30 as well as limits of detection mostly below 1 µg L^−1^ were achieved. These LOD values are, in principle, suitable for the analysis of contaminated waters, while a significant improvement in sensitivity by MS detection would be required for waters of low contamination degree, as in this study. Comparing our results to similar natured, previously reported methodologies, comparable or better analytical performance was achieved, with particularly high reproducibility and using a fully automated analyzer system. Running sample preparation and analyte separation in parallel, high time efficiency was achieved. The methodology is principally extendable to other analytes and matrices.

## Figures and Tables

**Table 1:** Summary of method performance for the evaluated five NSAIDs.

Analytes	Ret. Time ± SD	Calibration Curve Slope (L/µg)	R^2^	CF	LOD (µg L^−1^)	LOQ (µg L^−1^)	Repeatability, RSD (n = 6, 20 µg L^−1^) (%)	Inter-Day Precision, RSD (n = 12, 20 µg/L) (%)
KET	4.59 ± 0.03	981.15 ± 46.6	0.9983	15	0.62	1.9	4.2	7.9
NAP	5.01 ± 0.04	10982 ± 16.9	0.9975	14	0.06	0.2	3.2	6.0
FLB	8.91 ± 0.06	1120.4 ± 52.8	0.9955	26	0.88	2.7	3.9	5.7
DCF	10.93 ± 0.02	827.56 ± 8.9	0.9941	32	1.45	4.4	7.6	9.2
IBU	13.08 ± 0.08	680.28 ± 10.3	0.9978	19	1.98	6.0	3.6	5.2

CF—preconcentration factor in comparison with manual sample injection of 220 µL.

**Table 2:** Summary of sample analysis. Concentration levels given in (µg L^−1^), recovery given in (%). Concentration values ranging between calculated LOD and LOQ values are indicated in parentheses.

Analyte		KET	NAP	FLB	DCF	IBU
Sample 1	Genuine	<LOQ (1.1)	0.3	<LOD	<LOD	<LOD
	Added	20	20	20	20	20
	Found	21.1	20.3	20	19.4	21
	Recovery	106 ± 2	101 ± 1	100 ± 1	96.7 ± 3	105 ± 1
Sample 2	Genuine	<LOQ (1.1)	<LOD	<LOQ (0.3)	<LOD	<LOD
	Added	20	20	20	20	20
	Found	21.1	20	20.3	19.3	21.1
	Recovery	105 ± 2	100 ± 1	101 ± 1	96.4 ± 3	105 ± 1
Sample 3	Genuine	<LOQ (1.3)	0.6	<LOD	<LOD	<LOD
	Added	20	20	20	20	20
	Found	21.3	20.6	19.7	19.2	21.4
	Recovery	106 ± 2	103 ± 1	98.7 ± 1	95.9 ± 3	107 ± 1
Sample 4	Genuine	<LOQ (1.8)	0.3	<LOD	<LOD	<LOD
	Added	20	20	20	20	20
	Found	21.8	20.3	19.9	18.2	21.2
	Recovery	109 ± 2	101 ± 1	100 ± 1	90.9 ± 3	106 ± 1

## Data Availability

Not applicable.

## Supplementary Materials

The following items are available online, Figure S1: Comparison study of Phenomenex Strata-AX and Oasis HLB, Figure S2: Study of methanol content in eluent without and with the addition of ammonium hydroxide, Figure S3: Effect of methanol content in washing solution and effect of hydrochloric acid concentration in the sample before loading, Figure S4: Flow adapter for larger diameter microcolumns on a Lab-On-Valve port, Figure S5: Scheme of analogue circuit for PMW motor control, Figure S6: 3D printed stirring system for keeping the sorbent particles in suspension, Table S1: Summary of experimental parameters of the developed method and LIS–HPLC system, Table S2: Comparison with earlier reported methods for NSAIDs based equally on SPE and liquid chromatography with spectrophotometric detection, Table S3: Operation method for LIS–BI–HPLC, Table S4: Procedure to build column in the operation method “BuildColumn”, and Table S5: Procedure to discard sorbent in the operation procedure “DiscardSorbent”.

## Sample Availability

Samples of the compounds are not available from the authors.
