# Supplementary material for: Lab-In-Syringe with Bead Injection Coupled Online to High-Performance Liquid Chromatography as Versatile Tool for Determination of Nonsteroidal Anti-Inflammatory Drugs in Surface Waters
Source: Molecules. 2021 Sep 3;26(17):5358. doi: 10.3390/molecules26175358 (PMC8433787; doi:10.3390/molecules26175358)
Supplement: Supplementary file 1 [file molecules-26-05358-s001.zip › molecules-1366263-supplementary.pdf]

Supplementary Materials

Lab-In-Syringe with Bead-Injection coupled online to high performance liquid chromatography as versatile tool for determination of nonsteroidal anti-inflammatory drugs in surface waters

Celestine Vubangsi Gemuh, Burkhard Horstkotte, Petr Solich

Department of Analytical Chemistry, Faculty of Pharmacy in Hradec Králové, Charles University, Akademika Heyrovského, 1203, Hradec Králové 50 005, Czech Republic

Content:

Figure S1: Comparison study of Phenomenex Strata-AX and Oasis HLB.

Figure S2: Study of methanol content in eluent without and with the addition of ammonium hydroxide.

Figure S3: Effect of methanol content in washing solution and effect of hydrochloric acid concentration in the sample before loading.

Figure S4: Flow adapter for larger diameter microcolumns on a Lab-On-Valve port.

Figure S5: Scheme of analogue circuit for PMW motor control.

Figure S6: 3D printed stirring system for keeping the sorbent particles in suspension.

Table S1: Summary of experimental parameters of the developed method and LIS-HPLC system.

Table S2: Comparison with earlier reported methods for NSAIDs based equally on SPE and liquid chromatography with spectrophotometric detection.

Table S3: Operation method for LIS-BI-HPLC.

Table S4: Procedure to build column in the operation method "BuildColumn".

Table S5: Procedure to discard sorbent in the operation procedure "DiscardSorbent".

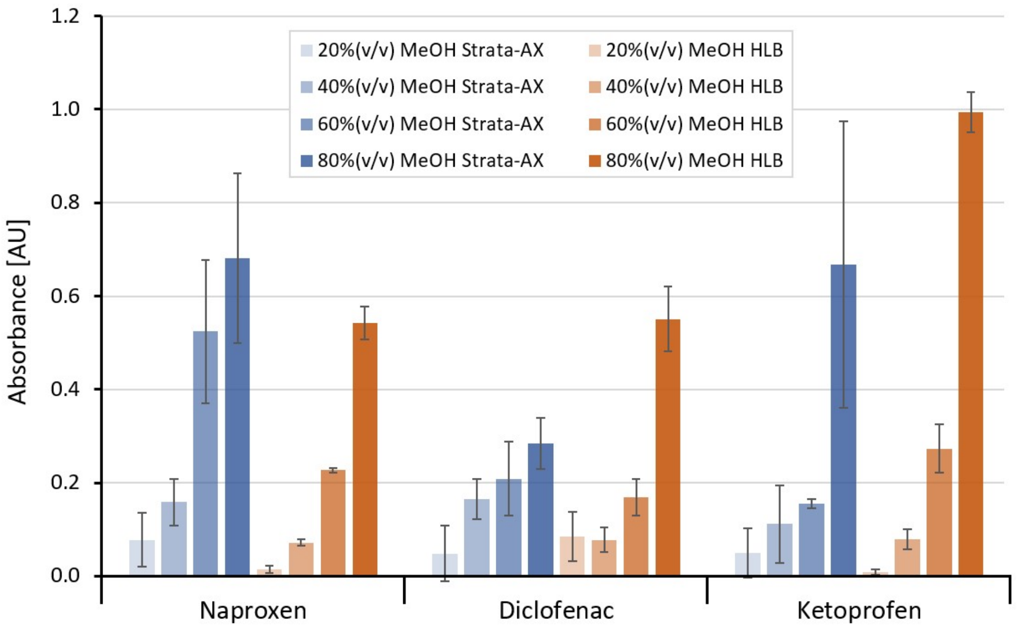

Figure S1: Comparison study of Phenomenex Strata-AX and Waters Oasis HLB resin for 3 NSAIDs using aqueous methanol mixtures of different concentrations as eluent. Elution peak heights were measured after BI in the flow system, i.e. not coupled to HPLC.

Conditions other than stated in section 2: Loading of 0.6 mL mixed 2 ppm standard, loading, washing, and elution flow rates = 15 µL s^{-1}, microcolumn of ca. 5 mg HLB sorbent, elution with 400 µL eluent. Strata-AX: sample alkalized with 200 µL of 100 mmol L^{-1} ammonium hydroxide. Addition of 10 mmol L^{-1} hydrochloric acid to the eluent. HLB: sample acidified with 200 µL of 100 mmol L^{-1} hydrochloric acid at 10 µL s^{-1}. Wavelengths = 230 nm (NAP), 250 nm (KET), 270 nm (DCF).

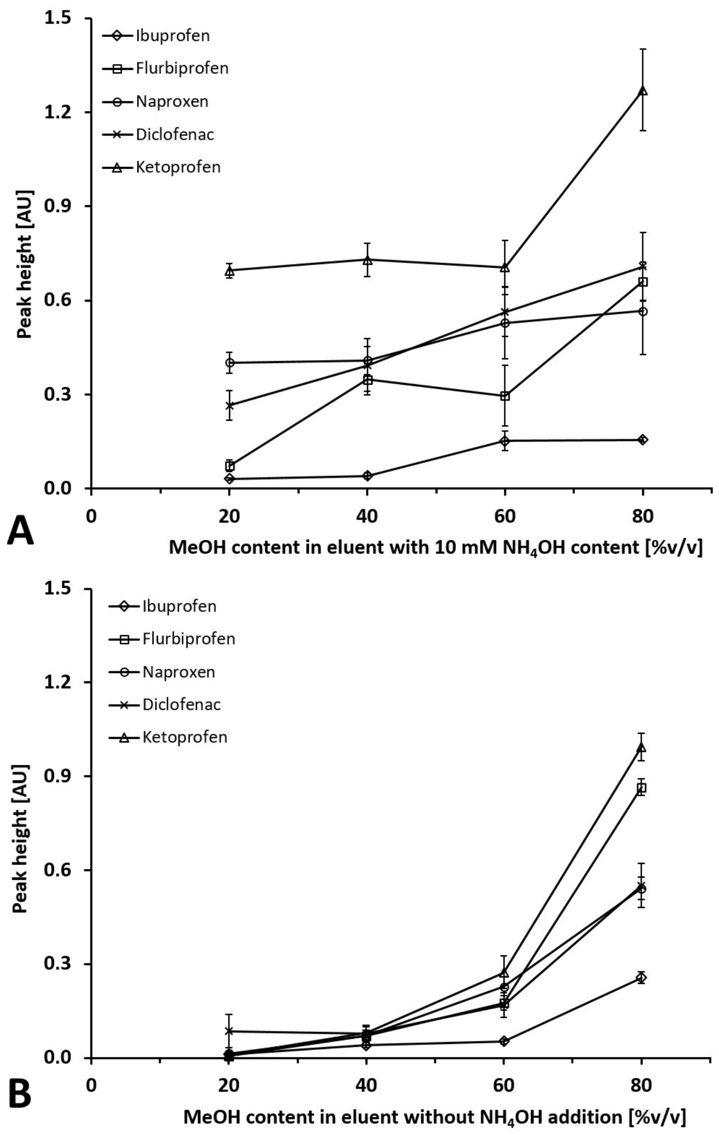

Figure S2: Study of methanol content in eluent (A) without and (B) with the addition of ammonium hydroxide by in-system spectrophotometric detection (no HPLC). Signals of NAP were scaled by factor 0.1. Conditions other than stated in section 2: Loading of 0.6 mL mixed 2 ppm standard acidified with 200 μL of 100 mmol L^{-1} HCl at 10 μL s^{-1}. Microcolumn of ca. 5 mg HLB sorbent, elution with 400 μL at 15 μL s^{-1} after sorbent washing with water. wavelengths = 230 nm (IBU and NAP), 250 nm (FLB and KET), 270 nm (DCF).

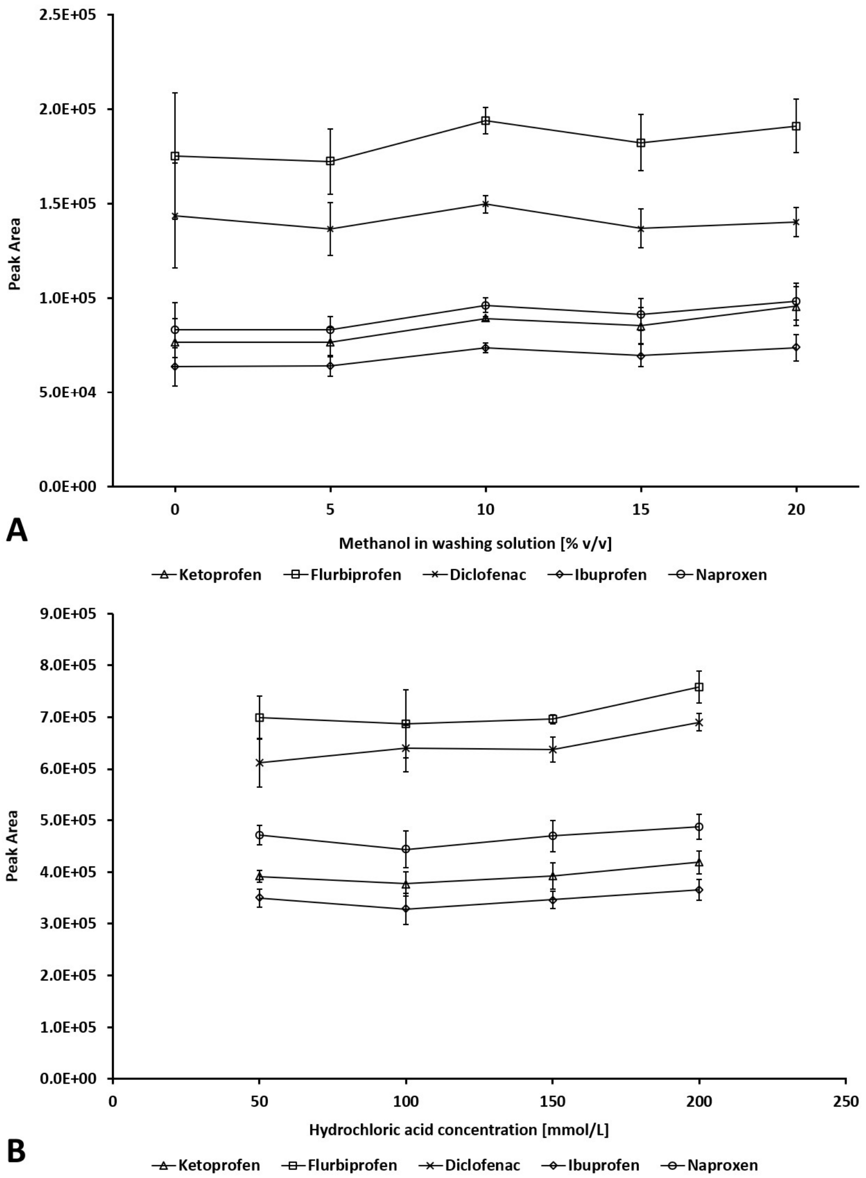

Figure S3: (A) Effect of methanol content in the washing solution and (B) effect of hydrochloric acid concentration in the sample before loading (B). Signals of NAP were scaled by factor 0.1. Conditions as given in Figure 2(D).

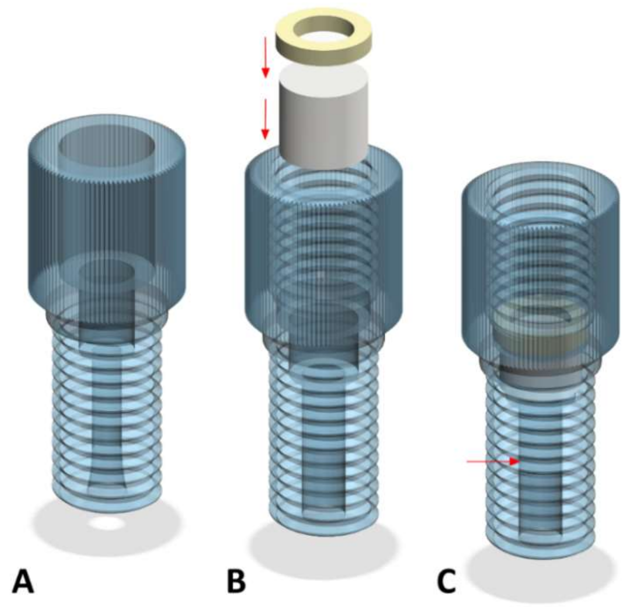

Figure S4: Flow adapter for larger diameter microcolumns on a Lab-On-Valve portIV . A: Commercial 1/8″ UNF fitting. B: modified with melamine foam and silicone rubber tube washer indicated, C: Assembled with indicated space for SPE microcolumn.

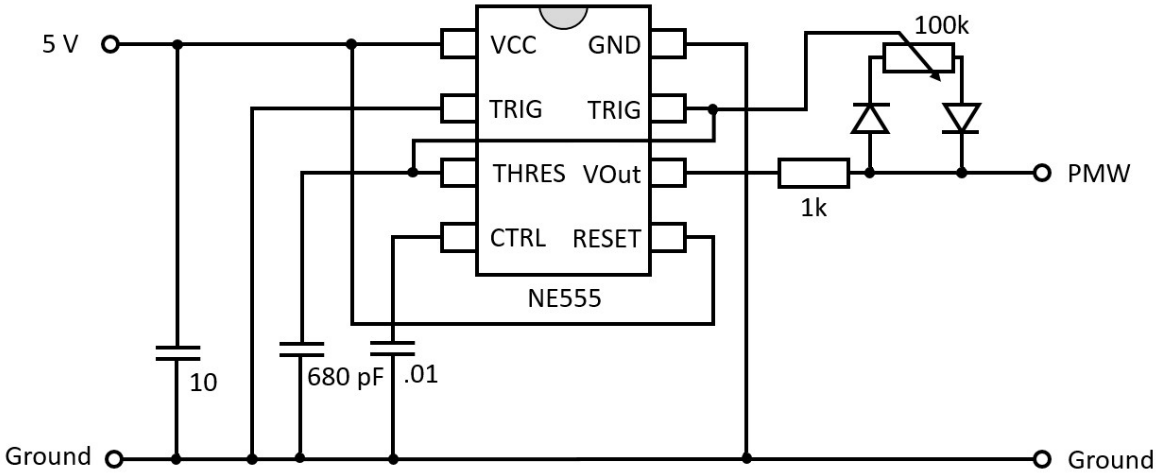

Figure S5: Scheme of analogue circuit for PMW motor control.

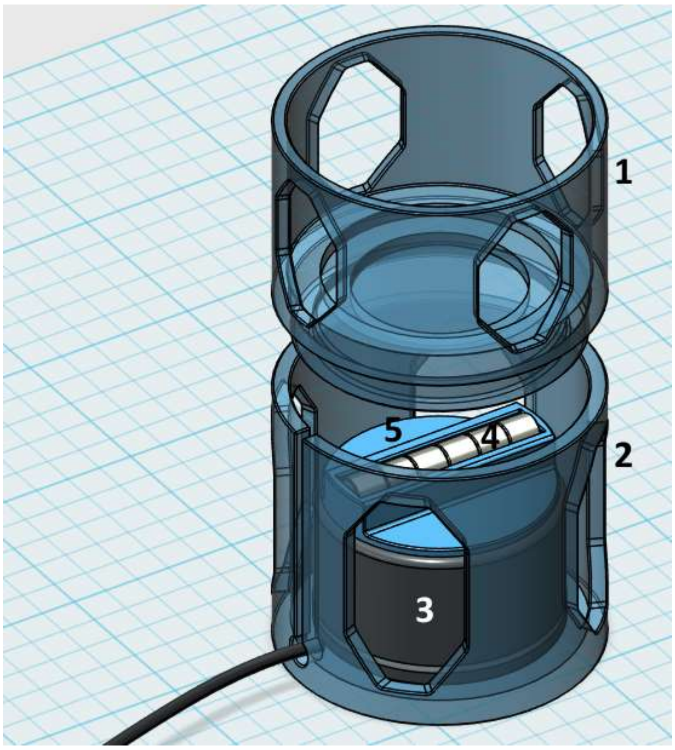

Figure S6: 3D printed stirring system for keeping the sorbent particles in suspension. 3D printed elements are shown in blue. Assembly of a vial compartment (1), a motor compartment (2), a DC motor featured from a 12 V computer fan (3), a USB cable, and neodymium magnets (4) fixed on top of the motor with a 3D printed holder (5). The top part intended to hold the vial with the sorbent suspension is lifted for clarity.

Table S1: Summary of experimental parameters of the developed method and LIS-HPLC system.

Parameters	Value
Loaded sample volume	3.8 mL
Added acid to sample	5 %(v/v) of 100 mol L^{-1} hydrochloric acid
Sorbent suspension	200 µL of 2.2 %(w/v) HLB corresponding to 4.4 mg sorbent
Washing solution	Water
Eluent	400 µL of 50%(v/v) acetonitrile
Applied flow rates for loading, washing, and elution	15 µL s^{-1}
Transfer volume (Volume of eluent pushed through microcolumn for loading)	350 µL
Injection loop	220 µL, 0.5 mm i.d.
Separation column	Symmetry C18 5 µm 4.6 x 150 mm
Mobile phase	40:60 %(v/v) ammonium formate buffer, pH 3.5 : ACN/MeOH
Flow rate HPLC	1.2 mL min^{-1}

Table S2: Comparison with earlier reported methods for NSAIDs based equally on SPE and liquid chromatography with spectrophotometric detection.

Extraction technique *	Sorbent *	Analytes	Sample type	Volume of sample [mL]	Linear range up to [µg L-1]	LOD [µg L-1]	SPE / LC [min]	Enrichment factor	Automation / Online *	Ref.
SPE	MIPs	KET, NAP, DCF, IBU`	Surface- & wastewaters	1-50	50 000	300-400	n.g. / 12	n.g.	no	[15]
SPE	Sol-gel	DCF, KET, MFA	River-, drinking, & tap waters	20	550	0.1-0.29	n.g. / > 6.5	400	no	[21]
µSPE	Ni-Fe LDH nanostructures	DCF, IGU, NAP, MFA	Urine	0.25	1 000	1-10	n.g/16	11.3	no	[24]
SBSE	Sol-gel	KET, FLB, NAP	Lake waters	10	500	0.23-0.31	45 / 12	51.6-56.3	no	[18]
dSPD	Magnetic particles	IBU, DCF, NAP, APAP	Wastewaters	1000	700	1-2	30 / 30	n.g.	no	[16]
dSPE	Magnetic graphene composite	KET, FLB, DCF, NAP, FEN	Plasma, urine	5	25 000	0.61-1.2	5 / 5	n.g.	no	[17]
SPME	Magnetic MOF fibers	IBU, DCF, NAP, NAL	Urine, serum, plasma	5	400	0.03-0.05	55 / 20	343-416	no	[22]
SPME	Polymeric IL-coated monolith	KET, FLB, DCF	Lake water, plasma	2	50	0.01-0.05	17 / 20	100	yes	[20]
RDSE	C18 sorbent disk	KET, NAP, DCF, IBU	Urine	10	2 000	22-44	15 / 14	17	yes	[19]
µSPE	Oasis HLB	KET, NAP, DCF, IBU, BZF	Surface- & wastewaters	18	40	0.02-0.36	20 / 16	100	Yes (BI)	[28]
µSPE	Oasis HLB	DCF, FLB, KET, NAP, IBU	Surface waters	4	200	0.06-1.98	10 / 13	14-32 **	Yes (BI)	This work

* Abbreviations other than used in the text: APAP: acetaminophen, BZF: bezafibrate, dSPE: dispersive solid-phase extraction, FEN: Fenbufen, IL: Ionic liquid, Nalidixic acid, LDH: layered double hydroxide, MFA: Mefenamic acid, MIPs: molecular imprinted polymers, NAL: Nalidixic acid, RSDE : rotating disk sorptive extraction, SBSE : stir bar sorptive extraction, SPME: Solid-phase microextraction. ** Considering 220 mL of injected sample.

Table S3: Operation method for LIS-BI-HPLC.

Operation	Comment
BUILD COLUMN	
Routine call ("BuildColumn")	Building the micro-column
Activate in-syringe stirring<br>Pickup 600 µL at 300 µL s^{-1} from LOV position II<br>Deactivate in-syringe stirring	Aspirate water for cleaning syringe
Pickup 300 µL at 150 µL s^{-1} from HV position 7<br>Wait 2 s	Aspirate the eluent
Empty syringe content at 15 µL s^{-1} through LOV position IV<br>Wait 3 s	Cleaning of microcolumn
ASPIRATION OF SAMPLE AND BUFFER	
Pickup 1000 µL at 200 µL s^{-1} from HV position 5<br>Wait 1 s	Aspiration of sample
Activate in-syringe stirring<br>Pickup 200 µL at 200 µL s^{-1} from HV position 8<br>Deactivate in-syringe stirring<br>Wait 1 s	Aspiration of hydrochloric acid
Dispense 400 µL at 200 µL s^{-1} through LOV position I<br>Wait 5 s	Filling the holding coil
Dispense 800 µL at 15 µL s^{-1} through LOV positionIV<br>Wait 30 s	Loading microcolumn
Empty syringe at 200 µL s^{-1} to LOV position V	Cleaning HC and pressure release
CLEANING OF SYRINGE	
Activate in-syringe stirring<br>Pickup 300 µL at 200 µL s^{-1} from HV position 3<br>Deactivate in-syringe stirring	Cleaning with 50 %(v/v) isopropanol
Empty syringe pump at 200 µL s^{-1} to HV position 1	Emptying syringe
Pickup 300 µL at 200 µL s^{-1} from HV position 2<br>Wait 3 s<br>Deactivate in-syringe stirring	Syringe cleaning with water
Empty syringe pump at 200 µL s^{-1} to HV position 1	Emptying syringe
Pickup 600 µL at 200 µL s^{-1} from HV position 2<br>Wait 1 s	Refilling syringe
WASHING THE MICROCOLUMN	
Pickup 300 µL at 200 µL s^{-1} from LOV position VI<br>Wait 2 s	Aspiration of washing solution
Dispense 400 µL at 15 µL s^{-1} through LOV channel IV<br>Wait 30 s	Washing the microcolumn
Empty syringe at 200 µL s^{-1} to LOV position V<br>Wait 1 s	Releasing the pressure
ELUTION	
Pickup 400 µL at 150 µL s^{-1} from LOV position VII<br>Wait 2 s	Aspiration of eluent
SWITCHING TO LOAD POSITION	
Dispense 350 µL at 15 µL s^{-1} through LOV position IV<br>Wait 30 s	Eluting sample into injection valve
Activate automatic injection<br>Switch to inject position	Injecting eluate onto HPLC column
Empty syringe pump at 15 µL s^{-1} through LOV position IV<br>Wait 600 s	Eluting remaining analytes and emptying syringe
DISCHARGE MICROCOLUMN	
Routine_call("DiscardSorbent")	Discarding the microcolumn

Table S4: Procedure to build column in the operation method "BuildColumn".

Operation	Comment
Pickup 1500 µL at 200 µL s^{-1} from HV position 2	Aspiration of water into the syringe
Dispense 500 µL at 200 µL s^{-1} to HV position 9 (LOV in position I)	Cleaning the holding coil
Pickup 200 µL at 200 µL s^{-1} from LOV position V	Aspiration of air
Dispense 200 µL at 200 µL s^{-1} to LOV position III<br>Wait 1 s	Pushing back remains of settled bead suspension into reservoir to ensure suspension homogeneity and high repoducibity of the next step
Pickup 200 µL at 200 µL s^{-1} from LOV position III<br>Wait 2 s	Aspiration of sorbent suspension
Dispense 500 µL at 20 µL s^{-1} to LOV position IV<br>Wait 10 s	Building the microcolumn from the beads contained in the aspirated bead suspension.<br>Waiting for allowing pressure release
Empty syringe at 200 µL s^{-1} through LOV position I<br>Wait 2 s	Emptying of syringe to waste

Table S5: Procedure to discard sorbent in the operation procedure "DiscardSorbent".

Operation	Comment
Pickup 600 µL at 200 µL s^{-1} from HV position 2<br>Wait 2 s	Aspiration of water
Pickup 400 µL at 200 µL s^{-1} from HV position 4<br>Wait 2 s	Aspirate 50 %(v/v) isopropanol
Dispense 400 µL at 15 µL s^{-1} to LOV position IV<br>Wait 10 s	Flushing the microcolumn
Pickup 50 µL at 50 µL s^{-1} from LOV position V<br>Re-aspirate trapped sorbent beads	Aspirate air to prevent beads entering syringe
Pickup 400 µL at 200 µL s^{-1} from LOV position IV<br>Wait 3 s	Aspiration of beads from channel
Empty syringe at 300 µL s^{-1} through LOV position I<br>Wait 2 s	Discharge beads to waste
